# Evaluation of effects of *Mycoplasma* mastitis on milk composition in dairy cattle from South Australia

**DOI:** 10.1186/s12917-017-1274-2

**Published:** 2017-11-25

**Authors:** Abd Al-Bar Al-Farha, Farhid Hemmatzadeh, Manouchehr Khazandi, Andrew Hoare, Kiro Petrovski

**Affiliations:** 1 School of Animal and Veterinary Sciences, The University of Adelaide, Roseworthy, South Australia 5371 Australia; 2 Northern Technical University, Technical Foundation, Mosul, Iraq; 3 Australian Centre for Antimicrobial Resistance Ecology, The University of Adelaide, Adelaide, South Australia 5000 Australia; 4 Davies Centre, School of Animal and Veterinary Sciences, The University of Adelaide, Roseworthy, South Australia 5371 Australia; 5 South East Vets, Mt Gambier, South Australia 5290 Australia

**Keywords:** *Mycoplasma*, Mastitis, Dairy cattle, Milk composition, Somatic cell count (SCC)

## Abstract

**Background:**

*Mycoplasma* mastitis is increasingly posing significant impact on dairy industry. Although the effects of major conventional mastitis pathogens on milk components has been widely addressed in the literature, limited data on the effects of different *Mycoplasma* and *Acholeplasma* spp. on milk quality and quantity is available. The aim of this study was to determine the casual relationship of *Mycoplasma* spp. and *A. laidlawii* to mastitis and compare them to subclinical mastitis caused by conventional mastitis pathogens from a single dairy herd in South Australia; *Mycoplasma* spp. and *A. laidlawii* were detected using PCR applied directly to milk samples. The herd had mastitis problem with high somatic cell count and low response rate to conventional antimicrobial therapy. A total of 288 cow-level milk samples were collected aseptically and used in this study.

**Results:**

Conventional culture showed a predominance of coagulase-negative staphylococci, followed by coagulase-positive staphylococci, *Streptococcus* spp., *Enterococcus* spp., *E. coli*, and *Klebsiella* spp. PCR results showed a high prevalence of mycoplasmas (76.7%), including *A. laidlawii* (10.8%), *M. bovis* (6.2%), *M. bovirhinis* (5.6%), *M. arginini* (2%), and (52.1%) of cows were co-infected with two or more *Mycoplasma* and *Acholeplasma* species. *Mycoplasma* co-infection significantly increased somatic cell counts (SCC) similar to conventional mastitis pathogens and compared to non-infected cows with 389.3, 550.3 and 67.3 respectively; and decreased the milk yield with 29.0, 29.9 and 34.4 l, respectively. *Mycoplasma* co-infection caused significant increase in protein percentage, and significant decrease in fat percentage and total milk solids, similar to other conventional mastitis pathogens. In contrast, changes in milk composition and yield caused by various individual *Mycoplasma* species were non-significant.

**Conclusions:**

*Mycoplasma* mastitis had on-farm economic consequences similar to common conventional mastitis pathogens. Results of our study indicate that co-infection *Mycoplasma* mastitis caused similar effect on milk composition to other mastitis pathogens and we hope these findings raise the awareness of the importance of their detection on routine diagnostic panels.

## Background

The genus *Mycoplasma* belongs to the class *Mollicutes* and is responsible for many diseases in cattle, including respiratory disorders, arthritis, otitis media, and mastitis [12, 23, 41]. *Mycoplasma* mastitis is highly resilient to antimicrobial therapy and can be easily missed during laboratory culture and susceptibility testing diagnostic panels [25]. Among the 200 species of *Mycoplasma* discovered to date, several have been reported to be involved in bovine mastitis such as *M. bovis*, *M. bovigenitalium, M. californium, M. bovirhinis, M. arginini, M. dispar, M. canadense, M. bovoculi*, and *Mycoplasma* spp. bovine groups 7 and F-38 [13]. Some studies have also indicated that *Acholeplasma* spp. can be a milk contaminant or non-pathogenic saprophyte [5, 27]. However, others have reported isolation of *A. laidlawii* from clinical and subclinical cases of bovine mastitis, suggesting a causal relationship [31, 61, 63].

In dairy herds, mycoplasmas can cause clinical, subclinical or chronic mastitis [16]. *M. bovis* is considered the most common pathogen among mycoplasmas [14]. The possibility of isolating *Acholeplasma* spp. from mastitis cases is not excluded [8, 31, 61, 63]. The incubation period of *Mycoplasma* mastitis is 10–14 days [48], and shedding of the causative pathogen may occur during this period contributing to the spread of bacteria. Economic consequences of *Mycoplasma* mastitis in cattle are due to decreased milk production, cost of implementing control procedures, and cost of diagnosis and treatment [39]. For instance, the cost of *M. bovis* infection in cattle is more than US$140 million annually in the United States, and even higher losses have been reported in Europe [2].

Bacteriological culture of mycoplasmas from milk samples was once the most common method of detection. However, this method is relatively slow often taking one to 2 weeks with potential non-growth of these bacteria due to their fastidious culture requirements [45, 51], *Mycoplasma* mastitis is usually excluded from general mastitis screening tests due to its special growth requirements and time delay [30]. Similarly, serological detection method is time-consuming as antibody formation requires approximately 2 weeks [26]. Furthermore, there is a variation in the growth requirements of different species of *Mycoplasma* [20] which consequently affects disease detection, particularly when *Mycoplasma* co-infection occurs. However, most mastitis diagnostic investigations focus on the predominant species of mycoplasma associated with infection, *M. bovis,* and disregard other causative *Mollicutes* [52]. Studies of clinical *Mycoplasma* co-infection also deserve more attention, especially for epidemiological and treatment investigations. Therefore, development of a rapid and reliable screening diagnostic method is required which will be capable in distinguishing between different mycoplasma genera and species.

The association between *Mycoplasma* mastitis, individual somatic cell count (SCC) and milk yield also requires clarification. The association between conventional pathogens causing mastitis, such as *Streptococcus* and *Staphylococcus* spp., and elevated SCC has been previously reported [10, 19]. *Mycoplasma* mastitis can also affect SCC patterns [32, 47]. A marked decrease in milk production has been estimated particularly from mastitis caused by *S. agalactiae*, *Mycoplasma* spp. and *Pasteurella* spp. [62]. However, the effect of *Mycoplasma* mastitis compared to conventional bacterial pathogens on other milk composition has yet to be evaluated. Furthermore, the pathogenicity of each individual *Mycoplasma* spp., either as a single or co-infection, needs to be explored.

This study had two aims. The first aim of this study was to determine the effects of different *Mycoplasma* spp. and *A. laidlawii* compared to conventional mastitis pathogens on milk yield and other milk components in cattle with high SCC (subclinical mastitis) from a single dairy herd in South Australia with low response rate to conventional antimicrobial therapy. The second aim was to identify *Mycoplasma* spp. and *A. laidlawii* using novel PCR applied directly to milk samples.

## Methods

### Sample collection

Milk samples were collected aseptically once from each individual cow from a single commercial dairy farm near Mount Gambier, South Australia. This farm had a history of repeated failure of mastitis treatment with high SCC. Samples originated from cows aged 2–10 years in the hospital mob or main milking mob if they had a high SCC. Composite milk samples were collected aseptically in sterile 50 mL tubes from each functional quarter of individual cows (*n* = 288). Samples were kept on ice and were sent immediately to the PC2 laboratory at The University of Adelaide, Roseworthy Campus. In the laboratory, milk samples were subjected to conventional microbial culture using 10 μL aliquots according to National Mastitis Council guidelines [24]. All samples were then frozen at −20 °C for further analysis.

### Milk analysis data

A database of individual cow yield production parameters (yield, total milk solids, fat and protein percentage) and SCC for sampled and non-sampled cows (the rest of the herd’s cows that have also high and low SCC) was obtained from herd testing information for the 4 herd tests closest to the sampling points (*n* = 7609 cow data points). SCC was performed for each milk sample using a FOSS Fossomatic 5000 (Hillerød, Denmark), and instrumental milk components assay by a commercial laboratory (NHIS, Cohuna, VIC, Australia).

### DNA extraction

After thawing milk at ambient temperature, 2 mL of each sample was centrifuged at 8000 *X* g for 20 min to remove supernatant fat and excess liquid. DNA extraction was performed directly from milk using the QIAmp DNA extraction kit (Qiagen, Germany) according to the manufacturer’s instructions with minor modifications. In order to increase the DNA yield, a much larger milk sample (1 mL) was centrifuged at 5000 *X* g for 30 min and the lipid layer removed. Genomic DNA concentration was measured using Nanodrop 1000c (Thermofisher Scientific Inc., Waltham, MA, USA) and stored at −20 °C until further use.

### PCR probes and protocol

A modified PCR protocol using four pairs of species-specific primers previously published elsewhere was developed (Table 1). In-vitro amplification of DNA to detect *Mycoplasma* and *Acholeplasma* spp. was carried out in 25 μL volumes consisting of 0.25 μL Taq DNA polymerase, 5 μL of 5× reaction buffer (Biolab, UK), 1 μL forward primer, 1 μL reverse primer, 1 μL of template DNA and 16.75 μL of DEPC-treated water. Amplifications were performed for 35 PCR cycles using the T100™ Thermal Cycler (Biorad Australia) and consisted of pre-heating activation for 5 min at 95 °C, denaturation at 95 °C for 30 s, and annealing at 60 °C for *M. bovis* and A. *laidlawii,* 55 °C for *M. arginini* and 64 °C for *M. bovirhinis.* The final extension step was performed at 72 °C for 10 min. The PCR products were subjected to electrophoresis in 1.5% agarose gels and visualised by staining with Gel Red.

**Table 1:** Oligonucleotides primers for detection of mycoplasmas and *A. laidlawii* identified by conventional PCR

Primers	Sequencing 5′-3′	Species	Targets	Amplicon size (bp)	Ref
Mb-F	CTTGGATCAGTGGCTTCATTAGC	*M.bovis*	*Vsp*A gene	400	[1]
Mb-R	GTCATCATGCGGAATTCTTGGGT				
Arg-F	AGAGTTTGATCCTGGCTCAGGA	*M. arginini*	16S rRNA gene	449	[11]
Arg-R	TCAACCAGGTGTTCTTTCCC				
Mbv-F	GCTGATAGAGAGGTCTATCG	*M. bovirhinis*	16S rRNA gene	316	[34]
Mbv-R	ATTACTCGGGCAGTCTCC				
Acho-F	AGCCGGACTGAGAGGTCTAC	*A. lailawii*	16S rRNA gene	505	[11]
Acho-R	TAATCCTGTTTGCTCCCCAC				

### Statistical analysis

For statistical analysis individual cows were grouped as follows:

- Bacteriologically analysed milk samples (all with SCC of >250,000 cells/mL and assumed to have subclinical mastitis, *n* = 288): which were positive for pure *M. bovis* detection (GROUP 1, *n* = 11); positive for pure *A. laidlawi* detection (GROUP 2, n = 28); positive for pure *M. bovirhinis* detection (GROUP 3, *n* = 13); positive for pure *M. arginini* detection (*n* = 6); positive for *Mycoplasma* co-infection by two or more detected species (GROUP 4, *n* = 119); positive for conventional mastitis pathogens and *Mycoplasma* negative (GROUP 5, *n* = 58), and cows with positive *Mycoplasma/Acholeplasma* detection and positive on conventional mastitis culture (GROUP 6, *n* = 53).
- Milk samples not bacteriologically analysed: cows with SCC of >250,000 cells/mL – assumed to have subclinical mastitis but not sampled (GROUP 7, *n* = 1146); and cows with SCC of ≤250,000 cells/mL assumed to be non-affected by subclinical mastitis ‘healthy cows’, (GROUP 8, *n* = 6181).

Statistical analysis software (SAS) version 9.4 (Cary Inc., USA) was used to analyse data.

For assessment of the effect of subclinical mastitis (1) or no subclinical mastitis (0) on SCC, herd test data were log transformed (somatic cell score; SCS). Due to the small number of positive detections (n = 6), the effect of *M. arginini* on milk composition and yield was not estimated.

The effect of subclinical mastitis on the test-day geometric mean of SCC was estimated using LOGISTIC REGRESSION in PROC GLIMMIX of SAS. The values obtained by modelling for the SCS were back-transformed to obtain the geometric mean of SCC. The effect of subclinical mastitis on test-day milk production parameters (yield, fat, protein and total milk solids) with pathogen as fixed effect and a cow as a random effect, was estimated using ANOVA in PROC MIXED of SAS. The effect of age and stage of production (i.e. days in milk) were not included in the final analysis as the preliminary model demonstrated no significant effect. A statistical difference of *P* < 0.05 was set as significant and *P* < 0.001 was highly significant.

## Results

Results showed that 221 of the 288 milk samples (76.7%) collected from the dairy farm were positive for *Mycoplasma* spp. and *A. laidlawii*. Among these, *Mycoplasma* co-infection with two or more genera/species predominated (52.1%), followed by single infections with *A. laidlawii* (10.8%)*, M. bovis* (6.2%)*, M. bovirhinis* (5.6%)*,* and *M. arginini* (2%) (Table 2). Agarose gel electrophoresis for PCR products revealed amplicon sizes stated in (Table 1). Conventional culture for all milk samples, independently of *Mollucites* isolation, showed a predominance of coagulase-negative staphylococci (CoNS) (12.2%), and relatively low occurrence of coagulase-positive staphylococci (CoPS) (2.4%), *Streptococcus* spp. (2.1%), *Enterococcus* spp. (1.7%), *E. coli* (1.4%), and *Klebsiella* spp. (0.4%) (Table 3).

**Table 2:** Distribution of *Mycoplasma* spp. detected by PCR for various conventional mastitis culture as detected directly from milk samples

Conventional mastitis culture	*Mycoplasma* detected spp.						Total
	*A. laidlawii*	*M. bovis*	*M. bovirhinis*	*M. arginini*	Mixed	Negative	
Negative (%)	28 (9.7)	11 (3.8)	13 (4.5)	3 (1)	119 (41.3)	56 (19.4)	230 (79.9)
Positive (%)	3 (1)	7 (2.4)	3 (1)	3 (1)	31 (10.76)	11 (3.8)	58 (20.1)
Total (%)	31 (10.8)	18 (6.2)	16 (5.6)	6 (2)	150 (52.1)	67 (23.2)	288 (100)

**Table 3:** Prevalence of non-*Mycoplasma* bacteria isolated by conventional mastitis culture from 288 milk samples collected purposively to identify *Mycoplasma* infections

Species.	Frequency (%)
CoNS	35 (12.2)
CoPS	7 (2.4)
*Streptococcus*	6 (2.1)
*Enterococcus*	5 (1.7)
*E.coli*	4 (1.4)
*Klebsiella*	1 (0.4)
ND	230 (79.86)
Total	288 (100%)

Significant difference in test-day SCC was detected between groups (*Mycoplasma* co-infection; GROUP 4, conventional mastitis pathogens; GROUP 5, mycoplasmas and other pathogens; GROUP 6, and not tested high SCC cows; GROUP 7) and assumed healthy cows (low SCC; GROUP 8; *P* < 0.001) with 389.32, 550.26, 611, 960.7 and 67.33 SCCx10^3^ cell/mL respectively. The *Mycoplasma* co-infected cows (GROUP 4) showed significant difference of SCC from assumed healthy cows (GROUP 8) at 376.15 SCCx10^3^ cell/mL (*P* ≤ 0.05). However, no significant difference was observed between cows infected with *M. bovis* (GROUP 1) and *A. laidlawii* (GROUP 2) in comparison with assumed healthy cows (GROUP 8) with 255.09 and 216.14, SCCx10^3^ cell/mL respectively (*P* > 0.05). Correspondingly, test day milk yield was similarly affected significantly in *Mycoplasma* co-infection (GROUP 4), conventional mastitis pathogens (GROUP 5) and mycoplasmas and conventional pathogens (GROUP 6) in comparison with assumed healthy cows (GROUP 8) with 29.0, 29.9, 27.9 and 34.4 l, respectively (*P* < 0.001). In contrast, milk yield did not decline significantly in cows infected with *M. bovis* (GROUP 1), *A. laidlawii* (GROUP 2), or *M. bovirhinis* (GROUP 3) with 32.4, 32.4 and 30.0 l/day, respectively (*P* > 0.05).

Fat percentage was significantly decreased in cows co-infected with *Mycoplasma* (GROUP 4) with 3.1% (*P* < 0.01), while no differences were observed in all other tested groups with 3.4, 3.3, 3.3, 3.2 and 3.3% for groups *M. bovis* (GROUP 1), *A. laidlawii* (GROUP 2), *M. bovirhinis* (GROUP 3), conventional mastitis pathogens (GROUP 5) and mixed *Mycoplasma/Acholeplasma* and conventional mastitis culture (GROUP 6) respectively (*P* > 0.05). Protein percentage increased significantly in cows with *Mycoplasma* co-infection (GROUP 4), conventional mastitis pathogens (GROUP 5), mixed *Mycoplasma/Acholeplasma* and conventional mastitis culture (GROUP 6) and non-tested high SCC (GROUP 7) in comparison with cows assumed to be healthy (GROUP 8) with 3.4, 3.3, 3.4, 3.4 and 3.3%, respectively (*P* < 0.05), while other *Mycoplasma* groups showed no differences being 3.4, 3.4 and 3.3% for GROUPs s 1, 2 and 3 respectively (*P* > 0.05). In comparison, total milk solids decreased significantly in GROUPs 4, 5, 6 and 7 in comparison to GROUP 8 with 1.8, 1.9, 1.8, 2.0 and 2.2%, respectively (*P* < 0.001). However, there were no differences in total milk solids for GROUPs 1, 2 and 3 with 2.1, 2.1 and 2.0%, respectively (P > 0.05) (Table 4).

**Table 4:** The mean values (± SE) of SCC, milk yield, milk fat, protein and total solids for dairy cattle infected with *Mycoplasma* and other mastitis pathogens (in individual cow-testing points)

		Group	Frequency	Milk yield (litre/day)	Fat percentage	Protein percentage	Total milk solids	SCCx10^3^cell/mL
Tested	1	*Mycoplasma bovis*	11	32.4 ± 3.73	3.4 ± 0.26	3.4 ± 0.09	2.1 ± 0.20	255.1 ± 140.07
	2	*Acholeplasma laidlawii*	28	32.4 ± 2.33	3.3 ± 0.16	3.4 ± 0.06	2.1 ± 0.13	216.1 ± 87.79
	3	*M. bovirhinis*	13	30.0 ± 3.43^a^	3.3 ± 0.24	3.3 ± 0.09	2.0 ± 0.19	376.2 ± 128.85^a^
	4	*Mycoplasma* co-infection	119	29.0 ± 1.13^A^	3.1 ± 0.08^a^	3.4 ± 0.03^A^	1.9 ± 0.06^A^	389.3 ± 42.58^A^
	5	Conventional mastitis pathogens	58	29.9 ± 1.57^a^	3.2 ± 0.11	3.3 ± 0.04^a^	1.9 ± 0.09^A^	550.3 ± 59.00^A^
	6	Mycoplasmas and conventional pathogens	53	27.9 ± 1.79^A^	3.3 ± 0.12	3.4 ± 0.04^A^	1.8 ± 0.11^A^	611 ± 12.08^A^
Total			282 (3.7%)					
Non tested	7	High SCC	1146	29.2 ± 0.36^A^	3.6 ± 0.02^A^	3.4 ± 0.01^A^	2.0 ± 0.02^A^	960.7 ± 13.72^A^
	8	Low SCC	6181	34.4 ± 0.15	3.4 ± 0.01	3.3 ± 0.00	2.2 ± 0.01	67.3 ± 5.90
Total			7327 (96.3%)					

Lower case superscripts indicate statistical difference within column of *P* < 0.05 or capital superscripts indicate statistical difference within column of *P* < 0.001

## Discussion

Results showed a high prevalence of *Mycoplasma* and *Acoleplasma* species isolated from the purposive sampled cows. In addition, *Mycoplasma* co-infection significantly changed milk quality and quantity, similarly to other mastitis pathogens. Details on the results of culture and PCR, including test characteristics, were not subject of this study and are not further detailed.

The study farm had a history of mastitis treatment failure and high SCC. The long persistence of this problem may be due to the variety of transmission methods, such as via direct contact, milking machines and other fomites [29]. Intermittent shedding of the pathogen from cows suffering chronic mastitis may be another important reason for the relatively high prevalence of *Mycoplasma* mastitis in the studied herd. Chronic mastitis results from the capability of *Mycoplasma* spp. to form multiple micro-abscesses within the infected mammary gland [28]. Due to the high occurrence of mastitis, the studied farm is not representative of the South Australian dairy cattle population. As the primary objective of this study was not to carry out an epidemiological investigation, but to purposively sample and collect a significant number of isolates for research, the validity of the study is not decreased. In addition, study results may raise awareness of the importance of *Mycoplasma* mastitis to the dairy industry. In order to establish the prevalence of this disease in Australia, further attempts in affected herds using methodology similar to that described in this study are required. It is possible that the PCR technique used in this study detects foreign pieces of DNA. To ensure validity of our results we also carried out conventional culture for *Mycoplasma* (data not shown). Indeed, some culture-negative samples yielded positive PCR. To ensure that we were detecting *Mycoplasma* spp. and *A. laidlawii,* 16S rRNA sequencing was also carried out (data not shown).

Results of this study highlight the tendency of *Mycoplasma* co-infection to cause a significant alteration in milk composition in comparison to any individual *Mycoplasma* spp. group. This seems to be a result of developing evidence for pathogenicity of *Mycoplasma* co-infection [17], and can be a reflection of the advanced stage of the disease. It is often thought that the mechanism of milk alteration is attributed to the pathogen severity, proportion of involved mammary glands alveoli, interference with blood or hormonal nourishment to these alveoli, epithelial integrity disruption, and milk decomposition due to enzymatic activity.

Differences between the effects of each individual *Mycoplasma* spp. and *A. laidlawii* on milk composition in this study is noteworthy. It has been reported worldwide that *M. bovis* is the primary contagious pathogen in bovine *Mycoplasma* mastitis [14, 35, 39, 43]. In our study, cows infected only with *M. bovis* (GROUP 1) showed no significant impacts on milk composition apart from the SCC. Cows in this group may have been at an early stage of the disease. In addition, the limited sample size of this group (*n* = 11) needs to be considered. However, the effect of *M. bovis* was significant when it contributed to the *Mycoplasma* co-infection group. Multiple *Mycoplasma* spp. infection tends to be more common in *Mycoplasma*-associated diseases [59]. Although, some studies indicate the possibility of *A. laidlawii* being involves in mastitis cases [8, 31, 63], our data showed no effect on milk composition as an individual pathogen, being similar to common veterinary literature that establishes these bacteria as a milk contaminant [4, 27, 40]. We considered increased SCC and decreased milk production in affected cows as a crucial factor confirming the contribution of *Mycoplasma* in bovine mastitis.

*M. arginini* was another species detected in this study. However, we excluded it from the comparison as an individual group due to the limited number of detection in milk samples (*n* = 6). *M. arginini* has been reported elsewhere with no significant impact on milk composition, but can be considered a predisposing agent to *Str. dysagalactiae* leading to severe mammary gland inflammation [58]. It is important to consider that *M. arginini* is associated with some diseases in human [56, 64]. Evidence of transmission of *M. arginini* to human through bovine milk is available as it has been isolated from blood of a man with a history of milk product consumption who had eosinophilic ascites for 2 years [57]. *M. bovirhinis* (GROUP 3) showed remarkable effect on SCC and milk yield in our study. *M. bovirhinis* was first isolated in mastitis cows in England [21] and named by Leach [37]. These bacteria showed predominance to other mycoplasmas in some mastitis studies [22, 50]. SCC can be elevated in cows inoculated experimentally with four different strains of *M. bovirhinis* causing subclinical mastitis [6]. However, the study of Brownlie et al. [6] lacks information on other milk composition changes.

Somatic cells mainly include macrophages, lymphocytes, polymorphonuclear and epithelial cells [44]. The elevation of these cells in bovine quarters reflects the possibility of infection and is the standard method to discriminate between healthy and mastitic cows [7, 42, 46, 54]. The acceptable limit of individual cow SCC in raw milk in Australia has been established at 250,000 cell/mL [9]. Elevated SCC appears to be an immune response to mastitis caused by *Mycoplasma*, often followed by spontaneous recovery [15]. The negative correlation between high SCC and low milk production has been widely accepted for mastitis cases. In this study, low milk yield also appeared to be associated with positive *Mycoplasma* infection which in turn was consistently associated with higher SCC, confirming the economic impact of mastitis caused by *Mycoplasma* mastitis. This decline in milk yield can be attributed to reduction of synthesis ability of the secretory mammary tissue [53].

The role of *Mycoplasma* mastitis in declining milk fat percentage during mastitis has not been established as yet. For other mastitis causing pathogens, contradictory results have been stated for both elevation [46, 55] and depression of milk fat percentage [3, 33, 53]. The decline in fat percentage seems to be a result of leukocyte ingestion to fat globule [49] or due to a decrease in the synthesis and secretion activities of mammary glands [36]. It is also important to note that variation of fat percentage can also affected by stage of lactation, genetics, management, nutrition and hormonal changes [38]. This most likely explains the elevation of fat percentage in the non-tested high SCC (GROUP 7) in this study. Our results show that milk protein percentage can be elevated significantly during mastitis caused by *Mycoplasma* co-infection (GROUP 5) or other non-*Mycoplasma* pathogens (GROUP 6). It is generally thought that milk proteins increase during mastitis due to increases in blood albumins and immunoglobulins influx as an immune response [3, 18]. Protein percentage increases have been reported during *M. agalactiae* experimental mastitis in small ruminant [60].

Decreases in total milk solids were tested to allow farmers, practitioners and scientists who deal with mastitis to directly compare effects of *Mycoplasma* infection on milk yield to each other and to other mastitis pathogens. SCC are usually available only at herd testing level, while total milk solids can be detected daily by in-line milk meters on some farms. Hence, the effect of different mastitis pathogens on total milk solids tends to be more important than SCC records to the current dairy industry. We are aware that SCC are more sensitive to detecting early intramammary infections. With time, in-line SCC measures may be available on many farms, and the importance of total milk solids may not be as high as at this stage. Data on decreases of total milk solids will also allow for further research into economic effects of *Mycoplasma* mastitis to a particular dairy herd or to the entire dairy industry.

Effects on milk production and SCC detected in our study may vary between farms and this should be an area of future research.

## Conclusions

In summary, we report bovine milk composition alteration during *Mycoplasma* mastitis. In addition, effects of *Mycoplasma* mastitis were compared with conventional mastitis pathogens. Results of our study indicate that co-infection *Mycoplasma* mastitis (GROUP 4) caused similar effect on milk composition to other conventional mastitis pathogens (GROUP 5), and we hope these findings raise the awareness of the importance of their detection on routine diagnostic panels. Ignoring timely detection may lead to developing *Mycoplasma* co-infection which may result in severe alterations in milk composition. However, roles of each individual *Mycoplasma* spp. in mastitis economics, and produced milk quantity and quality, needs further investigations, particularly when present as co-infections.

## Abbreviations

CoNS: Coagulase-negative staphylococci
CoPS: Coagulase-positive staphylococci
PCR: Polymerase chain reaction
SAS: Statistical analysis software
SCC: Somatic cell counts
SCS: Somatic cell scour
